# Supplementary material for: Phage-mediated peripheral kill-the-winner facilitates the maintenance of costly antibiotic resistance
Source: Nat Commun. 2025 Jul 1;16:5839. doi: 10.1038/s41467-025-61055-y (PMC12219745; doi:10.1038/s41467-025-61055-y)
Supplement: Supplementary file 1 — Supplementary Information [file 41467_2025_61055_MOESM1_ESM.pdf]

Supplementary Information for

Phage-mediated peripheral kill-the-winner facilitates the maintenance of costly antibiotic resistance

Chujin Ruan[1#*], Deepthi P. Vinod[1,2#], David R. Johnson[1,3*]

[1]Department of Environmental Microbiology, Swiss Federal Institute of Aquatic Science and Technology (Eawag), 8600 Dübendorf, Switzerland; [2]Department of Environmental Systems Science, Swiss Federal Institute of Technology (ETH), 8092 Zürich, Switzerland; [3]Institute of Ecology and Evolution, University of Bern, 3012 Bern, Switzerland. [#]These authors contributed equally.

[*]Correspondence:
David R. Johnson, david.johnson@eawag.ch; Chujin Ruan, chujin.ruan@eawag.ch

Contents:

Supplementary Table 1: Parameters used for the individual-based computational simulations.
Supplementary Fig. 1: Growth kinetics of strains AS and $AR_{C,Tet}$ and $AR_{C,Str}$ in batch culture.
Supplementary Fig. 2: Phage one-step growth assays in batch culture.
Supplementary Fig. 3: Fitness cost of antibiotic resistance determines the effect of phage lysis on the persistence of AR cells.
Supplementary Fig. 4: Fitness cost of antibiotic resistance and rate of phage lysis determine the persistence of slower-growing AR strains at a fixed total biomass size.
Supplementary Fig. 5: Effect of the fitness cost of antibiotic resistance and the rate of phage lysis on the persistence of AR cells.
Supplementary Fig. 6: Phage lysis increases the persistence of slower-growing AR cells in the face of spontaneously generated AS cells across different environmental conditions.
Supplementary Fig. 7: Properties of spontaneously emerging AS cells determine the persistence of slower-growing AR strains at a fixed total biomass size.
Supplementary Fig. 8: Faster-growing AS cells that emerge spontaneously are disproportionately lysed by phage.
Supplementary Fig. 9: Fitness cost of antibiotic resistance, rate of phage lysis, and probability of losing antibiotic resistance determine the total number of antibiotic resistance loss events.
Supplementary Fig. 10: Fitness cost of antibiotic resistance, rate of phage lysis, and probability of losing antibiotic resistance determine the removal of AS and AR cells.
Supplementary Fig. 11: Fitness cost of antibiotic resistance, rate of phage lysis, and probability of losing antibiotic resistance determine the removal of AS and AR cells.
Supplementary Fig. 12: Phage lysis maintains strain diversity.
Supplementary Fig. 13: Phage lysis maintains strain diversity when analyzing simulations at a fixed simulation time.

Supplementary Table 1: Parameters used for the individual-based computational simulations

Parameter	Description	Value	Unit
g_{AS}	Specific growth rate of an AS cell	1	-
g_{AR}	Specific growth rate of an AR cell	0.1-0.95	-
L_0	Mean initial length of a cell	2	µm
L_d	Length of a cell at division	3.5-4	µm
$w_{infection}$	Width of the outer cell layer affected by	4	µm
d_{cell}	Mean diameter of a cell	0.5	µm
N	Initial number of cells	2000	cell
R	Rate of phage lysis	0.01-0.05	-
P	Probability of losing antibiotic resistance	0.01-0.03	-

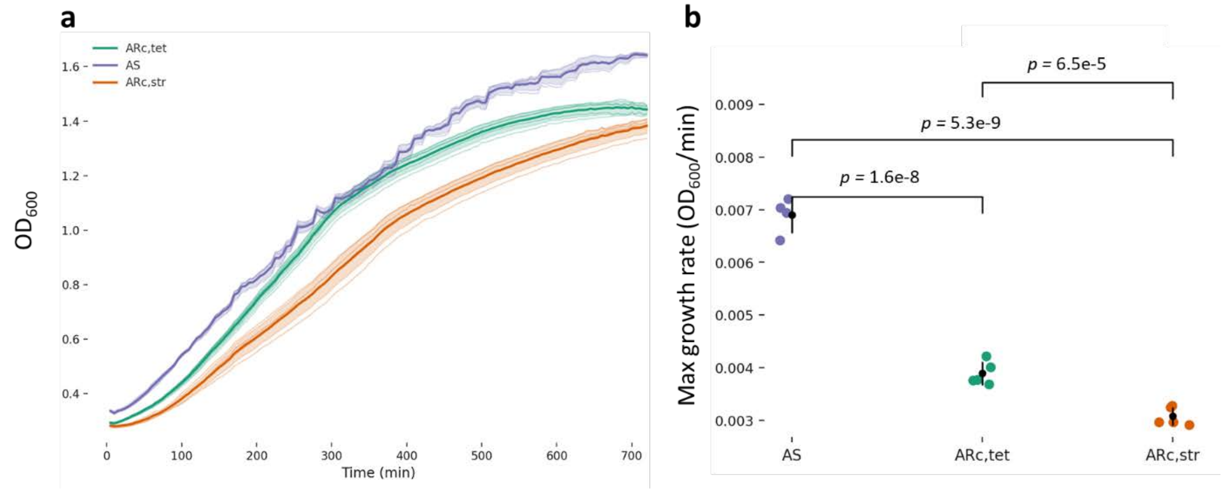

Supplementary Fig. 1: Growth kinetics of strains AS and $AR_{C,Tet}$ and $AR_{C,Str}$ in batch culture. a, Optical density at 600 nm (OD_{600}) measurements over time for strains AS, $AR_{C,Tet}$ and $AR_{C,Str}$ at 21°C in the absence of phage. Each line connects the mean values and the shaded region is ± one standard deviation from the mean (n = 5). **b**, Maximum growth rates of each strain in the absence of phage. Each colored data point is an independent experimental replicate. The black data point is the mean value and the black vertical line is ± one standard deviation from the mean (n = 5). The *p*-values are for two-sample two-sided Welch tests with a Bonferroni correction. Source data are provided as a Source Data file.

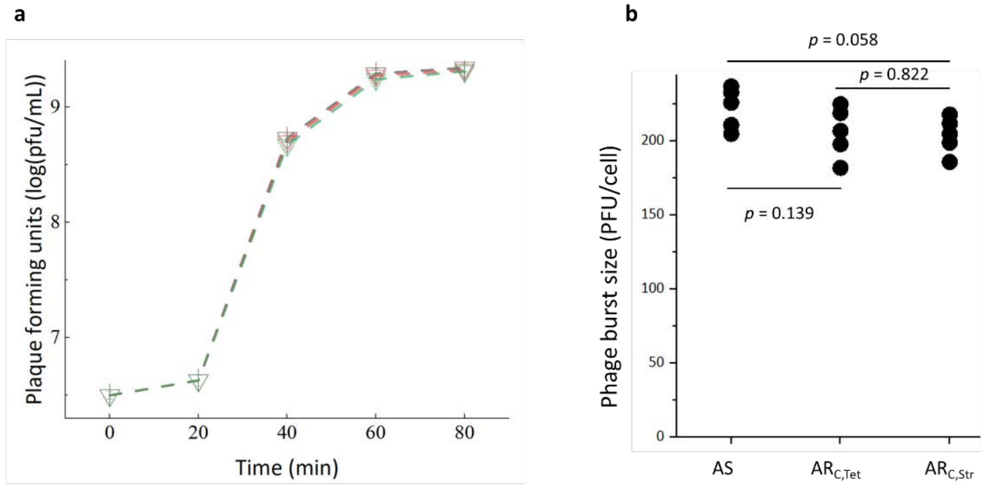

Supplementary Fig. 2: Phage one-step growth assays in batch culture. a, Numbers of plaque forming units (pfu/ml) estimated in an 80-minute period for strains AS, $AR_{C,Tet}$ and $AR_{C,Str}$. Each line connects the mean values and error bars are ± one standard deviation from the mean (n = 5). **b**, Burst sizes of phage for strains AS, $AR_{C,Tet}$ and $AR_{C,Str}$ determined from the phage growth curves. Each data point is an independent experimental replicate. The *p*-values are for two-sample two-sided Welch tests with a Bonferroni correction. Source data are provided as a Source Data file.

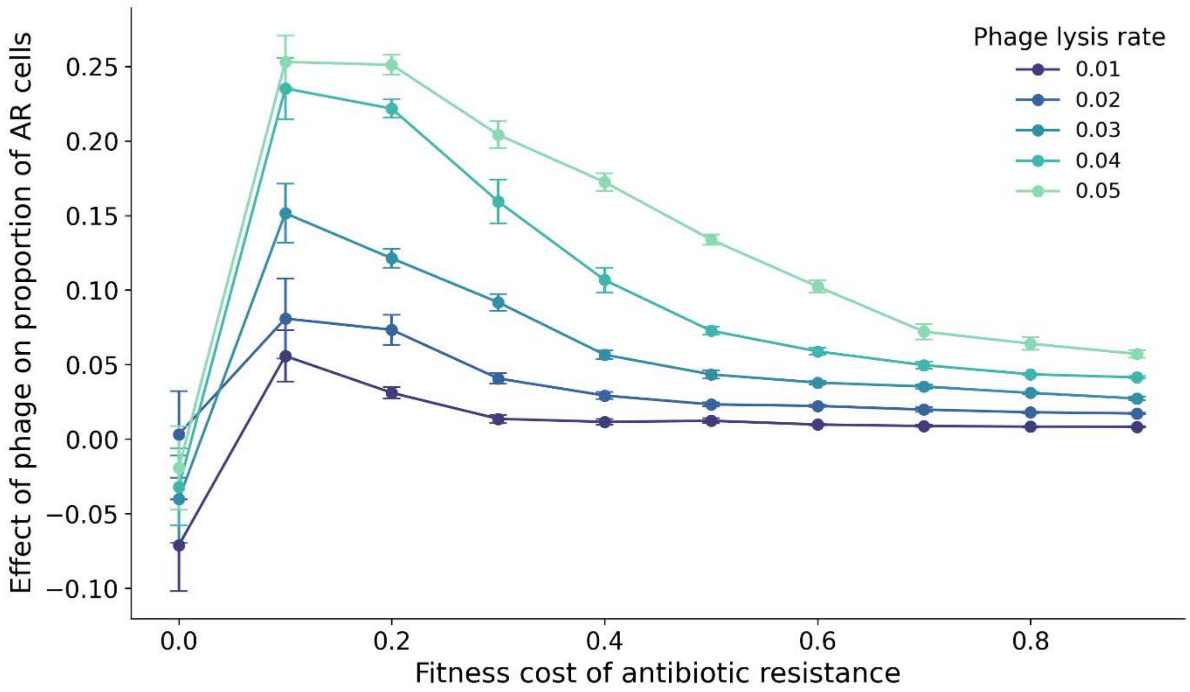

Supplementary Fig. 3: Fitness cost of antibiotic resistance determines the effect of phage lysis on the persistence of AR cells. The effect of phage on the proportion of AR cells is the proportion of AR cells in the presence of phage minus the proportion of AR cells in the absence of phage. All quantities are those at a fixed simulation time. Each data point is an independent simulation (n = 4), the lines connect the mean values, and the error bars are ± one standard deviation from the mean. Source data are provided as a Source Data file.

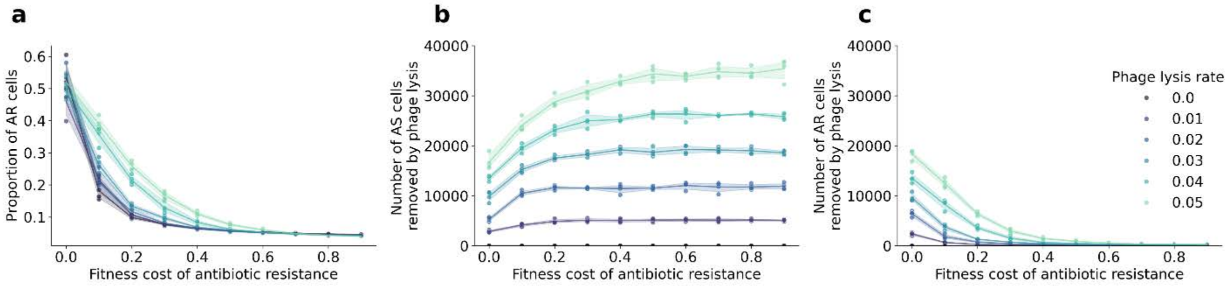

Supplementary Fig. 4: Fitness cost of antibiotic resistance and rate of phage lysis determine the persistence of slower-growing AR strains at a fixed total biomass size. All data are for individual-based computational simulations of co-cultures consisting of strains AS and AR with different fitness costs of antibiotic resistance and rates of phage lysis. **a**, The proportions of AR cells as a funcstion of the fitness cost of antibiotic resistance for different rates of phage lysis. **b**, The number of AS cells removed by phage as a function of the fitness cost of antibiotic resistance for different rates of phage lysis. **c**, The numbers of AR cells removed by phage as a function of the fitness cost of antibiotic resistance for different rates of phage lysis. For **a**–**c**, each data point is an independent simulation (n = 4), the lines connect the mean values, and the shaded regions are ± one standard deviation from the mean. Source data are provided as a Source Data file.

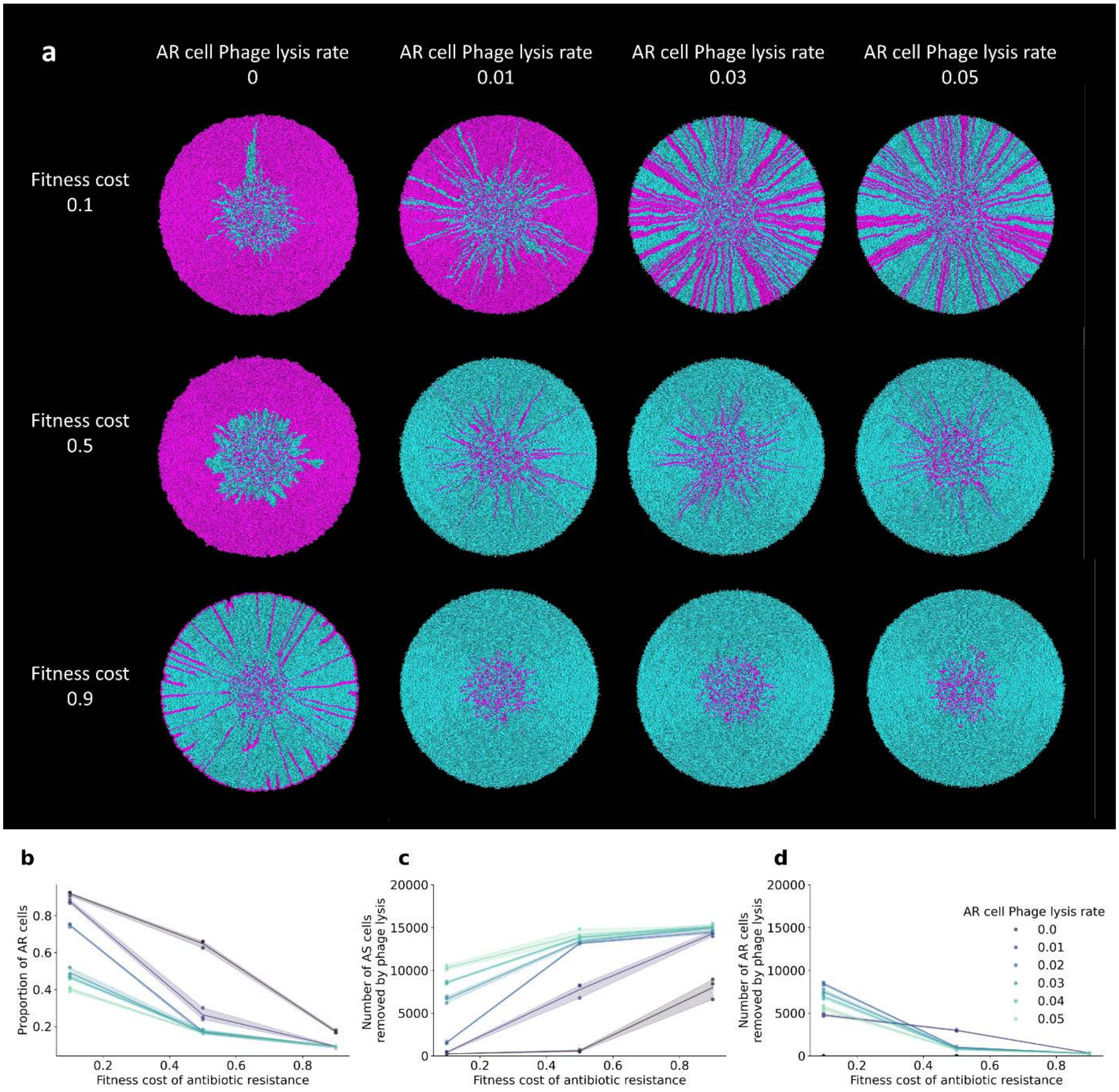

Supplementary Fig. 5: Effect of the fitness cost of antibiotic resistance and the rate of phage lysis on the persistence of AR cells. a, Representative individual-based computational simulations of co-cultures of faster-growing AS cells (cyan) and slower-growing AR cells (magenta) for different combinations of the fitness cost and rate of phage lysis for AR cells. The rate of phage lysis for AS cells was fixed at 0.05. **b**, The final proportions of AR cells as a function of the fitness cost for different rsates of phage lysis. **c**, The total numbers of AS cells removed by phage lysis as a function of the fitness cost and rate of phage lysis. **d**, The total numbers of AR cells removed by phage lysis. For **b**-**d**, each data point is an independent simulation (n = 3), the lines connect the mean values, and the shaded areas are ± one standard deviation from the mean. Source data are provided as a Source Data file.

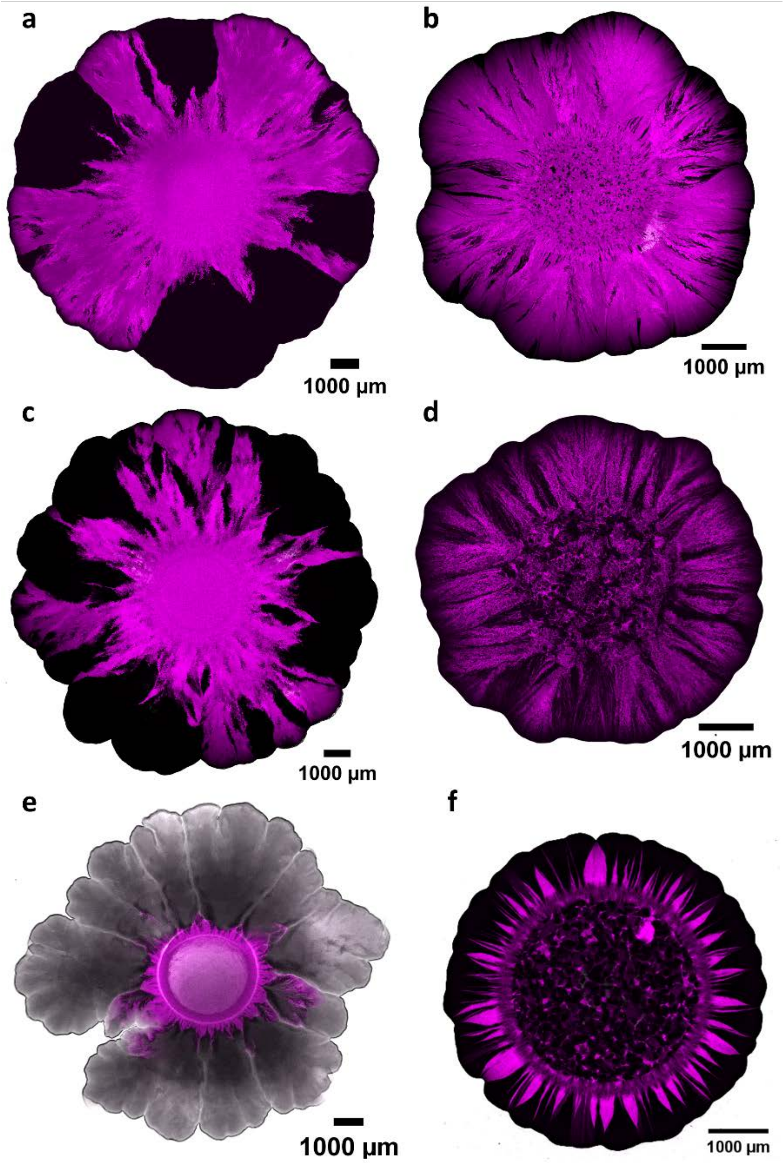

Supplementary Fig. 6: Phage lysis increases the persistence of slower-growing AR cells in the face of spontaneously generated AS cells across different environmental conditions. **a**,**b**, Representative CLSM images of strain $AR_{P,Chl}$ in the (**a**) absence or (**b**) presence of phage T6 after 16 days of growth in oxic conditions at 21°C in the absence of antibiotic pressure. **c**,**d**, Representative CLSM images of strain $AR_{P,Chl}$ in the (**c**) absence or (**d**) presence of phage T6 after ten days of growth in oxic conditions at 30°C in the absence of antibiotic pressure. **e**,**f**, Representative CLSM images of strain $AR_{P,Chl}$ in the (**e**) absence or (**f**) presence of phage T6 after ten days of growth in anoxic conditions at 21°C in the absence of antibiotic pressure. For all images, fluorescent (magenta) regions are $AR_{P,Chl}$ cells and non-fluorescent regions are $AR_{P,Chl}$ cells that lost plasmid pEF001 and became AS cells.

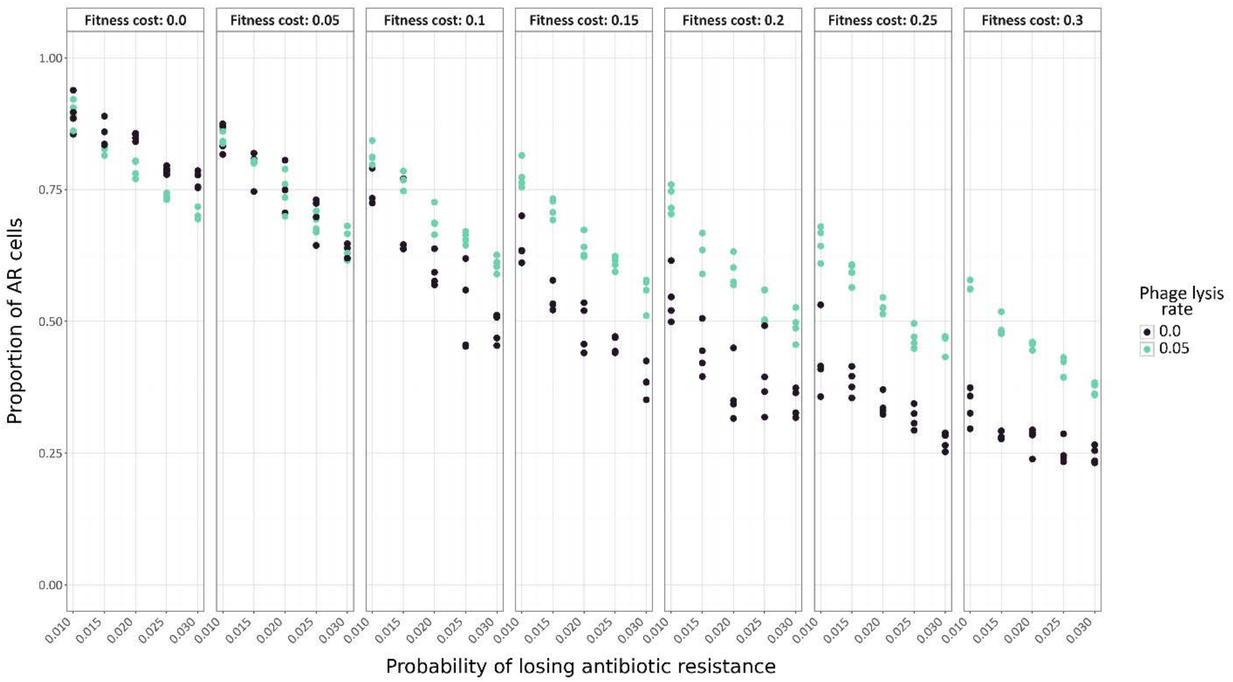

Supplementary Fig. 7: Properties of spontaneously emerging AS cells determine the persistence of slower-growing AR strains at a fixed total biomass size. All data are for individual-based computational simulations of strain AR in the absence or presence of phage lysis. The proportion of AR cells is calculated for different fitness costs of antibiotic resistance, probabilities of losing antibiotic resistance, and rates of phage lysis. Each data point is an independent simulation (n = 4), the black data points are in the absence of phage, and the green data points are in the presence of phage. Source data are provided as a Source Data file.

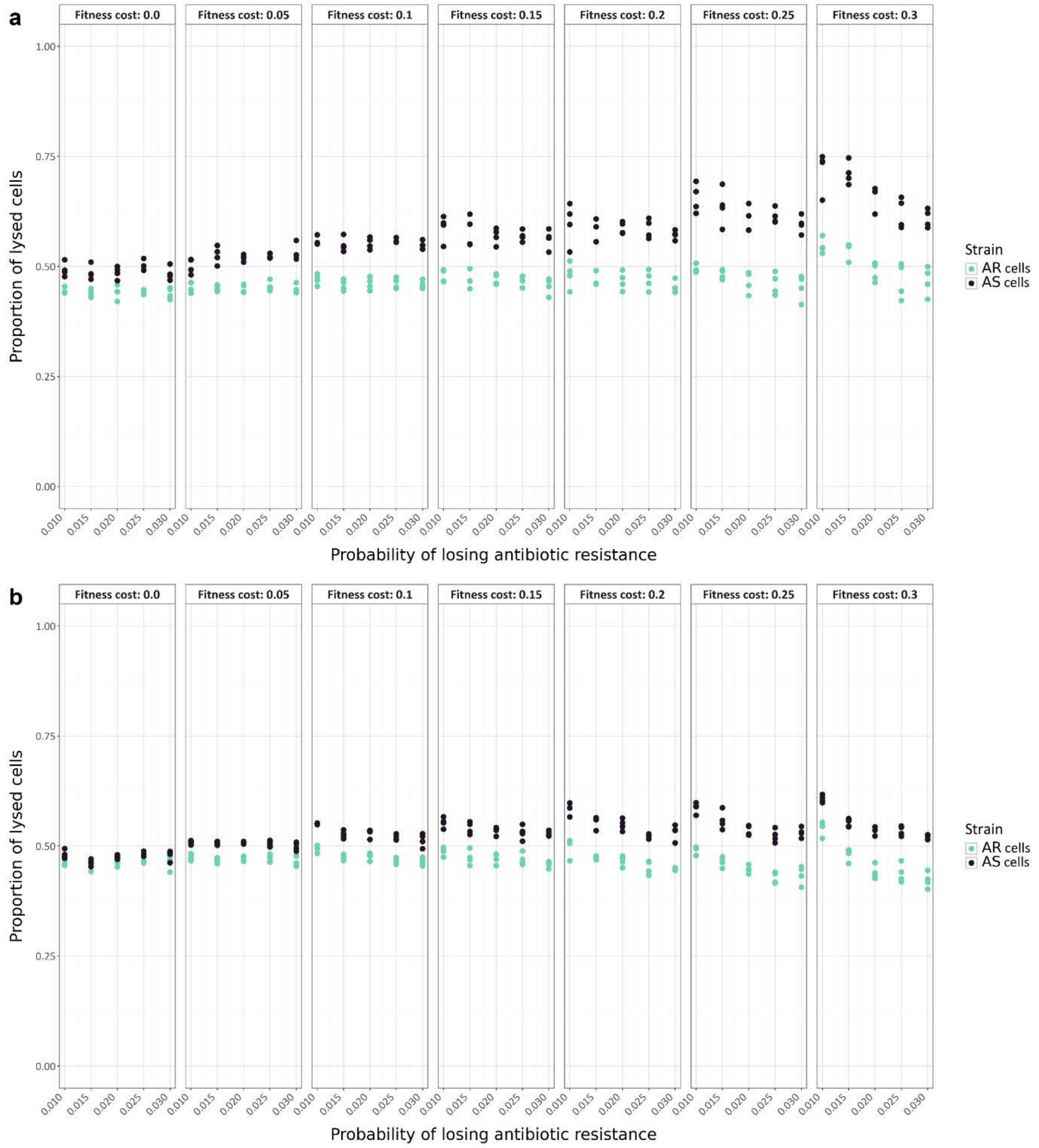

Supplementary Fig. 8: Faster-growing AS cells that emerge spontaneously are disproportionately lysed by phage. All data are for individual-based computational simulations of strain AR in the absence or presence of phage lysis. The proportions of lysed AS (black) and AR (green) cells are calculated for different fitness costs of antibiotic resistance and probabilities of losing antibiotic resistance. **a**,**b**, The proportions are the number of lysed cells of one strain to the total number of cells of that strain at (**a**) a fixed simulation time, or (**b**) a fixed total biomass size. Each data point is an independent simulation (n = 4), the black data points are in the absence of phage, and the green data points are in the presence of phage. Source data are provided as a Source Data file.

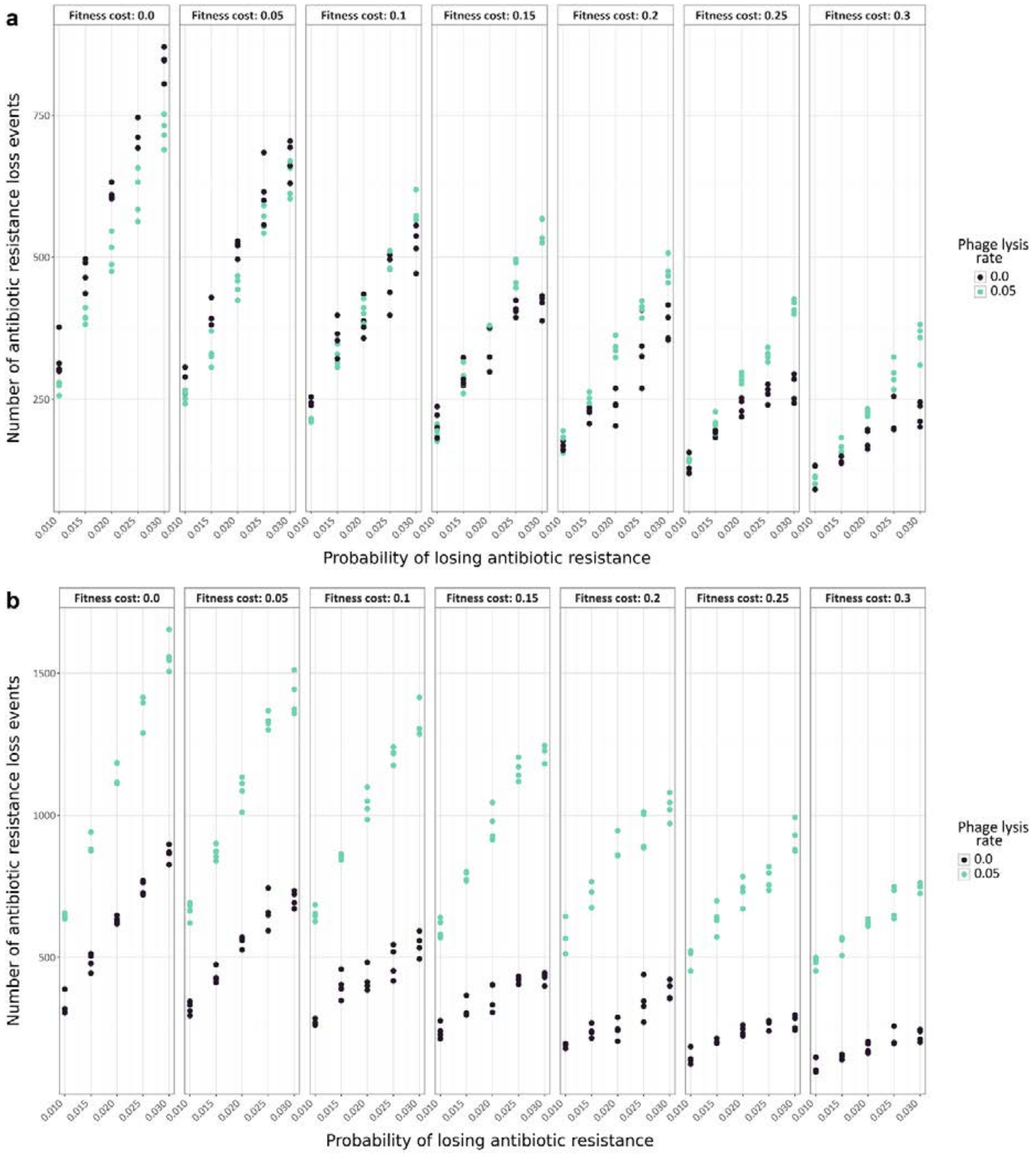

Supplementary Fig. 9: Fitness cost of antibiotic resistance, rate of phage lysis, and probability of losing antibiotic resistance determine the total number of antibiotic resistance loss events. **a**,**b**, All data are for individual-based computational simulations of strain AR for different fitness costs of antibiotic resistance and probabilities of losing antibiotic resistance in the absence or presence of phage at **(a)** a fixed simulation time, or **(b)** a fixed total biomass size. Each data point is an independent simulation (n = 4), the black data points are for a rate of phage lysis of zero, and the green data points are for a rate of phage lysis of 0.05. Source data are provided as a Source Data file.

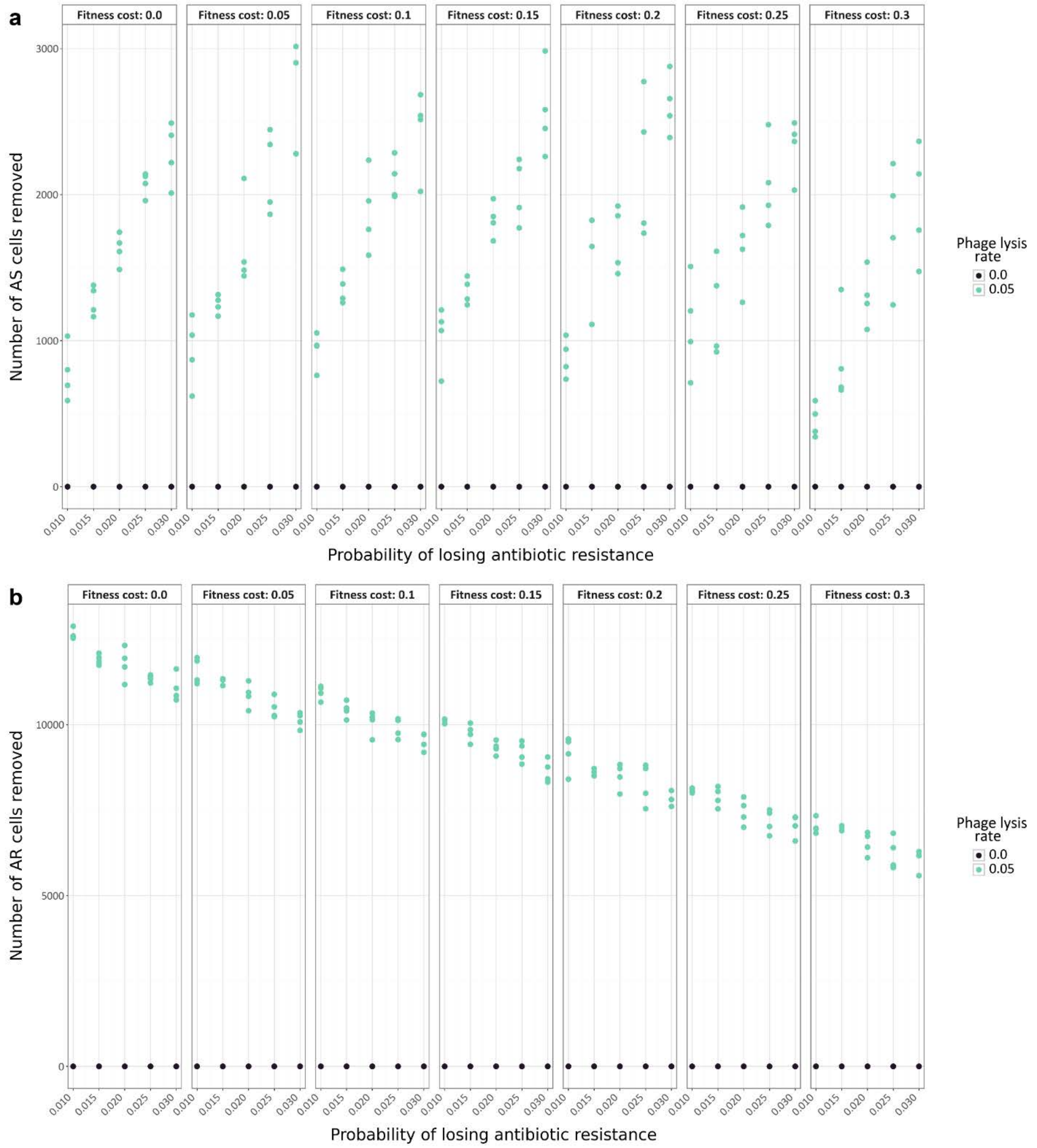

Supplementary Fig. 10: Fitness cost of antibiotic resistance, rate of phage lysis, and probability of losing antibiotic resistance determine the removal of AS and AR cells. a,**b**, All data are for individual-based computational simulations of strain AR in the absence or presence of phage at a fixed simulation time. **a**,**b**, The number of (**a**) AS cells or (**b**) AR cells removed by phage for different fitness costs of antibiotic resistance and probabilities of losing antibiotic resistance. For **a**,**b**, each data point is an independent simulation (n = 4), the black data points are for a rate of phage lysis of zero, and the green data points are for a rate of phage lysis of 0.05. Source data are provided as a Source Data file.

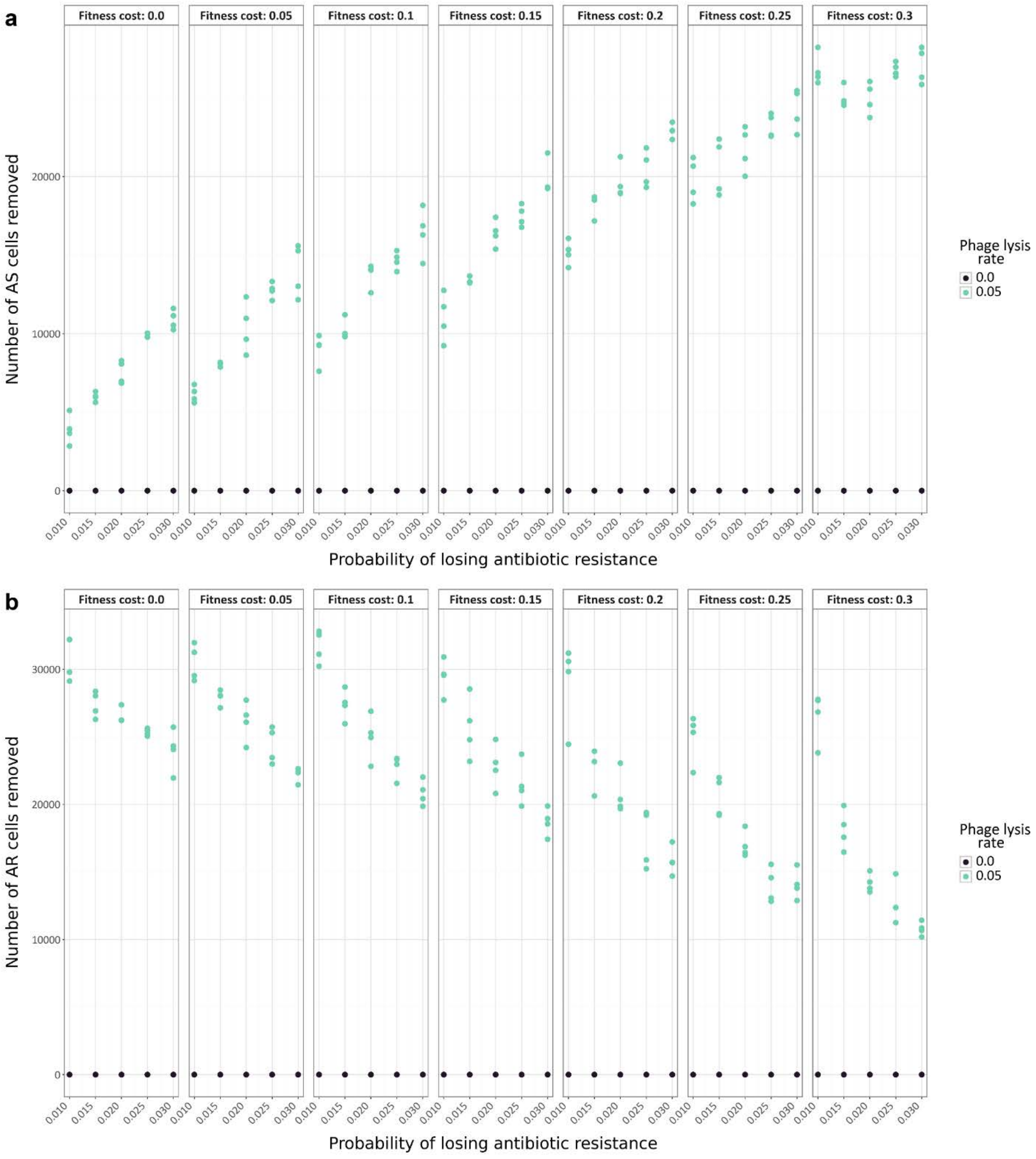

Supplementary Fig. 11: Fitness cost of antibiotic resistance, rate of phage lysis, and probability of losing antibiotic resistance determine the removal of AS and AR cells. All data are for individual-based computational simulations of strain AR in the absence or presence of phage at a fixed total biomass size. **a**,**b**, The number of (**a**) AS cells or (**b**) AR cells removed by phage for different fitness costs of antibiotic resistance and probabilities of losing antibiotic resistance. For **a**,**b**, each data point is an independent simulation (n = 4), the black data points are for a rate of phage lysis of zero, and the green data points are for a rate of phage lysis of 0.05. Source data are provided as a Source Data file.

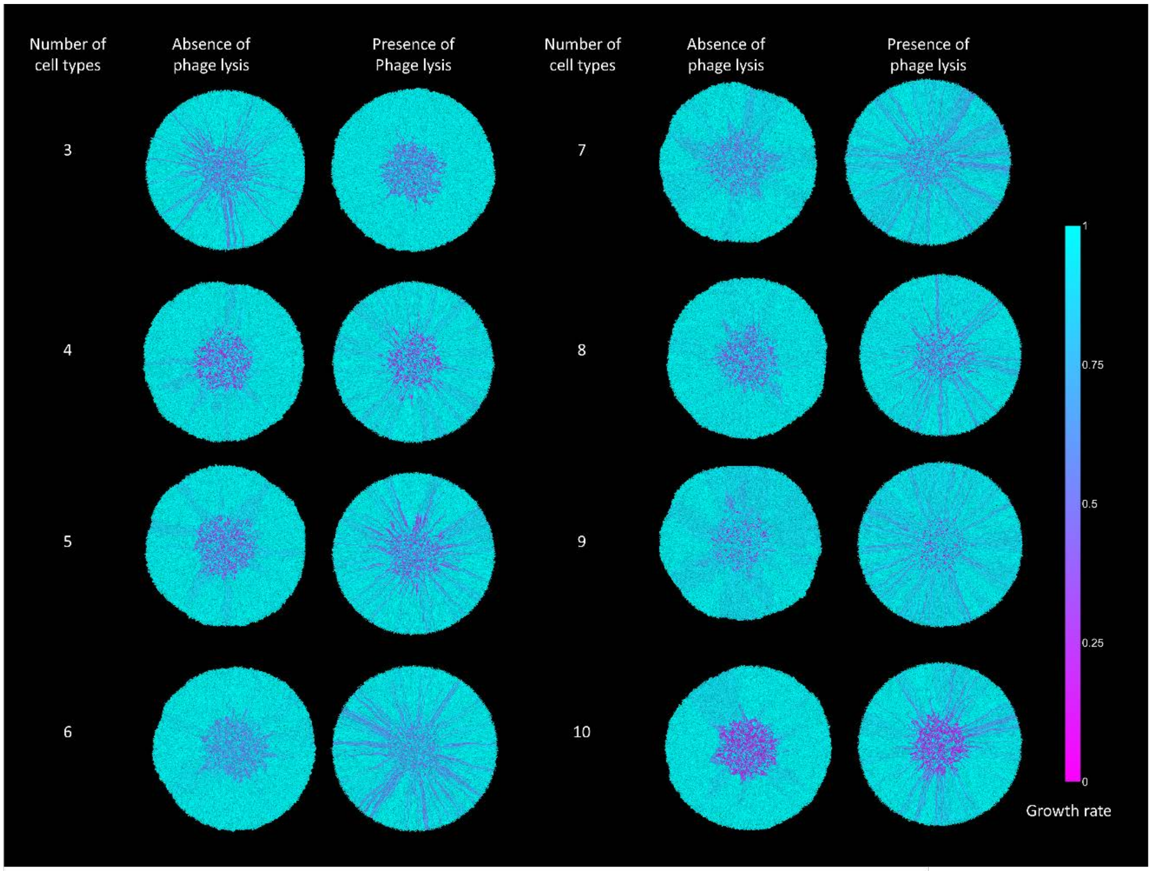

Supplementary Fig. 12: Phage lysis maintains strain diversity. Representative individual-based computational simulations of co-cultures consisting of two to ten distinct strains in the absence or presence of phage. The images are the outcomes at a fixed total biomass size. Each strain has a different growth rate ranging between zero and one that was randomly assigned by sampling from a uniform distribution. The color of each cell corresponds to its growth rate, represented as a gradient from magenta (growth rate = 0) to cyan (growth rate = 1).

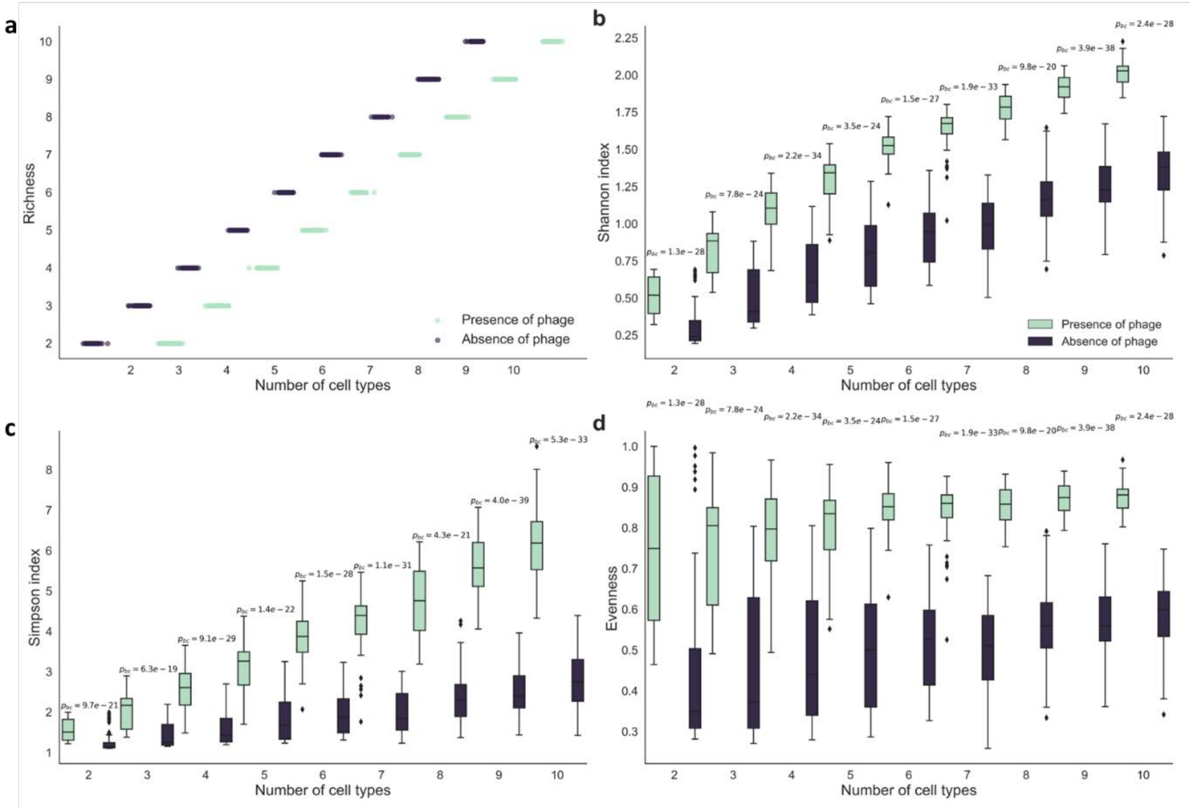

Supplementary Fig. 13: Phage lysis maintains strain diversity when analyzing simulations at a fixed simulation time. Data are for individual-based computational simulations of co-cultures of two to ten strains, where each strain has a different growth rate ranging between zero and one. The growth rate of one strain is one and the growth rates of the other strains are sampled from a uniform distribution of growth rates. **a**-**d**, The strain diversity metrics are those quantified at the last simulation time and include (**a**) strain richness, (**b**) Shannon diversity, (**c**) Simpson diversity, and (**d**) evenness. For **b**-**d**, the boxplots identify the mean values, interquartile ranges, and outliers for 473 independent pairs of simulations in total (between 35-65 replicates for any given number of cell types). The black boxplots and data points are in the absence of phage and the green boxplots and data points are in the presence of phage. The *p*-values are for two-sided paired t-tests with a Bonferroni correction. Source data are provided as a Source Data file.
