# Supplementary material for: Phage-mediated peripheral kill-the-winner facilitates the maintenance of costly antibiotic resistance
Source: Nat Commun. 2025 Jul 1;16:5839. doi: 10.1038/s41467-025-61055-y (PMC12219745; doi:10.1038/s41467-025-61055-y)
Supplement: Supplementary file 2 — Description Of Additional Supplementary Files [file 41467_2025_61055_MOESM2_ESM.docx]

File Name: Supplementary Movie 1

Description: Representative individual-based computational simulations of spatial patterns formed by co-cultures of strains AS and AR for different fitness costs of antibiotic resistance and rates of phage lysis. We initiated the simulations with 1,000 AS cells (cyan) and 1,000 AR cells (magenta) and performed simulations until the total number of cells reached 40,000. Simulations are for three different fitness costs of antibiotic resistance and four different rates of phage lysis.

File Name: Supplementary Movie 2

Description: Representative individual-based computational simulations of spatial patterns formed by the AR strain for different fitness costs of antibiotic resistance, probabilities of losing antibiotic resistance, and rates of phage lysis. The AR cells (magenta) have a fitness cost for antibiotic resistance. Each cell can stochastically transition into an AS cell (grey) with a certain probability, in which case it will be relieved of its fitness cost. We initiated the simulations with 2,000 AR cells and performed simulations until the total number of cells reached 40,000. Simulations are for three different fitness costs of antibiotic resistance and three different probabilities of losing antibiotic resistance. The upper simulations are with phage lysis and the lower simulations are without phage lysis.
